# Expression of Urea Transporter B in Normal and Injured Brain

**DOI:** 10.3389/fnana.2021.591726

**Published:** 2021-05-28

**Authors:** Boyue Huang, Hongkai Wang, Dandan Zhong, Jia Meng, Min Li, Baoxue Yang, Jianhua Ran

**Affiliations:** ^1^Department of Anatomy, Laboratory of Neuroscience and Tissue Engineering, Basic Medical College, Chongqing Medical University, Chongqing, China; ^2^Department of Pharmacology, School of Basic Medical Sciences, State Key Laboratory of Natural and Biomimetic Drugs, Peking University, Beijing, China

**Keywords:** ScRNA-seq, distribution, neuron, TBI, urea transporter B

## Abstract

Urea transporter B (UT-B) is a membrane channel protein widely distributed in mammals, and plays a significant physiological role by regulating urea and water transportation in different tissues. More and more studies have found that UT-B is related to neurological diseases, including myelinopathy and depression. When urea accumulates in the brains of UT-B knockout mice, the synaptic plasticity of neurons is reduced, and the morphology and function of glial cells are also changed. However, the distribution and expression change of UT-B remain unclear. The purpose of this study is to determine the expression characteristics of UT-B in the brain. Through single-cell RNA sequencing, UT-B was found to express universally and substantially throughout the various cells in the central nervous system except for endothelial and smooth muscle cells. UT-B was detected in the third cerebral ventricular wall, granule cell layer of the dentate gyrus, and other parts of the hippocampal, cerebral cortex, substantia nigra, habenular, and lateral hypothalamic nucleus by immunohistochemistry. Compared with the membrane expression of UT-B in glial cells, the subcellular localization of UT-B is in the Golgi apparatus of neurons. Further, the expression of UT-B was regulated by osmotic pressure *in vitro*. In the experimental traumatic brain injury model (TBI), the number of UT-B positive neurons near the ipsilateral cerebral cortex increased first and then decreased over time, peaking at the 24 h. We inferred that change in UT-B expression after the TBI was an adaptation to changed urea levels. The experimental data suggest that the UT-B may be a potential target for the treatment of TBI and white matter edema.

## Introduction

Urea, as the primary end product of protein catabolism in mammals, has a very high transporting rate in several kinds of mammalian cells. Urea transporters (UT) are a group of membrane channel proteins facilitating the massive and rapid movement of urea through different tissues (Sands, 1999). Urea transporter B (UT-B), encoded by the *Slc14a1* gene, has been documented in various tissues including kidney, ureter, bladder, testis, erythrocyte, fetal liver, spleen, bone marrow, intestine, colon, heart, aorta, cochlea, and brain (Sands and Blount, 2014). UT-B facilitates urea movement across lipid bilayers along a chemical gradient, which contributes to nitrogen homeostasis. In addition to urea permeability, UT-B can also transport water (Yang and Verkman, 1998; Huang et al., 2017) and a variety of urea analogs, including methyl urea and formamide (Yang, 2014).

The UT-B is widely distributed in the central nervous system. UT-B protein was found in astrocyte endfeet processes surrounding microvessels and ependymal cells lining along cerebral ventricles (Trinh-Trang-Tan et al., 2002). Urea produced by neurons is eliminated across UT-B in glial cells (Gilad and Gilad, 1992). UT-B was originally thought to be expressed only in glial cells and passively transports urea into the cerebrospinal fluid and blood, maintaining the steady-state of osmotic pressure and transporting the end products of amino acid metabolism in the brain. More and more studies have discovered the toxic effects of urea on the nervous system (Wang et al., 2014). As the only protein known to transport urea in the brain, UT-B has also attracted wide attention. UT-B deletion results in the accumulation of urea in the brain, causes nerve morphological changes, such as loss of neurons, swelling of nerve fibers and astrocytes, and membranous myelin-like structure formation within myelinated and unmyelinated fibers (Yang B. et al., 2014). Nuclear dissolution in neurons with normal perikaryon was also observed occasionally in UT-B-null mice. High urea concentration in the medial prefrontal cortex inhibited the mTORC1-S6K-dependent dendritic protein synthesis, impaired long-term potentiation in mPFC, which lead to depression-like behavior (Li et al., 2011).

Given the increased research on UT-B in the nervous system, investigations for the physiological role of UT-B in the brain are highly necessary. Finding out the location of UT-B in the brain is more conducive to understanding its function in the nervous system. Although the distribution of the UT-B mRNA in the central nervous system (Couriaud et al., 1996; Bagnasco, 2003; Stewart, 2011; Shayakul et al., 2013) has been revealed, the UT-B protein remains vague due to the possible rapid degradation or inefficient translation of the mRNA produced in abundance.

In this study, we used single-cell RNA sequencing (scRNA-seq) to determine the expression of UT-B in 4,884 cells from the wild-type mouse brain and analyzed the expression ratio of UT-B in each cell subtype. The distribution of UT-B in rat brain regions was detected by immunohistochemistry. Whether in the mouse, rat, or human brain, we confirmed the expression of UT-B in neurons. We also found the subcellular location and expression level of UT-B were regulated by osmotic pressure in cultured cortical neurons. UT-B expression level changed in the rat brain trauma over time. These data uncovered a detailed description of the UT-B expression in the central nervous system. Furthermore, the expression characteristics of UT-B regulated by osmotic pressure suggest that it may be involved in the pathological progression of brain edema and further research is needed to elucidate thoroughly.

## Methods

### Animals

Adult male Sprague-Dawley rats in the weight range of 220–250 g obtained from the Animal Breeding Facility of Chongqing Medical University were prepared for the experiments. Rats (n = 30) were divided into five groups based on time intervals after traumatic brain injury (normal control, 3, 6, 24, 72 h). Animal studies were conducted following the procedure approved by the Animal Ethics Committee of Peking University. The approval number is LA2020354.

C57BL/6J male and female mice (7- to 8-week-old) were acclimated to Peking University, Beijing, vivarium for at least 7 days before experiments. Food and water were available *ad libitum*. All protocols involving mice were approved by the Institutional Animal Care and Use Committee at the Peking University Health Science Center (Beijing, China).

UT-B knockout mice were generated by crossing C57BL/6J background germline-heterozygous-null mutant UT-B knockout mice as described (Yang et al., 2002). The offspring were genotyped by PCR using mouse-tail DNA and wild-type and mutant allele-specific primers (5′-AGGTGTGGCCTCAAAGTACTTGGCTA-3′). The PCR products were visualized with ethidium bromide staining.

#### Single-Cell RNA Sequencing

Artificial cerebrospinal fluid was prepared as follows: 87 mM NaCl, 2.5 mM KCl, 1.25 mM NaH_2_PO_4_, 26 mM NaHCO_3_, 75 mM sucrose, 20 mM glucose, 1 mM CaCl_2_, 7 mM MgSO_4_, adjusted to pH 7.4, equilibrated in 95% O_2_ and 5% CO_2_. Eight-week-old UT-B^−/−^ and UT/B^+/+^ mice were deeply anesthetized and perfused through the heart with cold artificial cerebrospinal fluid (ACSF) prepared previously. After taking the brain tissue out of the skull, a mouse brain slice mold was used to cut into 1-mm brain slices. The brain tissues were then digested using ACSF diluted papain at 37°C for 30 min and were mixed every 10 min After filtering with a 400-mesh cell sieve, the suspension was diluted with 50 ml ACSF at 4°C. After 30-min enzymatic digestion at 37°C with shaking of the tube every 10 min, the suspensions were filtered through an ACSF-equilibrated 35-mm cell strainer. After filtering, the suspension was diluted in a large-volume (50 ml total) ice-cold ACSF, followed by centrifugation (200 g, 5 min) to reduce debris. The supernatant is then carefully discarded. The pelleted cells were resuspended in 200 μl of dulbecco's modified eagle medium (DMEM) containing 1% bovine serum albumin (BSA). The 10 × genomics chromium single-cell kit was then used to perform scRNA-seq detection on the suspension.

### Traumatic Brain Injury Model

The experimental traumatic brain injury model was created in the right hemisphere referred to Feeney's method (Feeney et al., 1981) with heavier weight and higher height. After rats were deprived of water and food for 6 h, anesthesia was performed with pentobarbital (60 mg/kg) by intraperitoneal injection. The head was fixed in a stereotactic apparatus along the midline incision scalp, periosteal stripping exposed to the right parietal bone. The bone window of 5 mm in the diameter was created using a dental drill 3 mm behind the coronal slit and 3 mm beside the midline; the dura mater integrity was kept. A weight of 40 g fell freely from a height of 25 cm to hit and make a contusion of the right parietal lobe. Then the bone window was closed with the bone wax and after the bleeding was stopped, scalp incision was sutured with a drop of gentamycin. Except for the brain contusion impact, the same steps were repeated in the sham operation group. A bone window of the same size was established at the same position as the rats in the operation group. The window was covered with gauze moistened with saline. According to the time points after injury (3 h, 6 h, 24 h, and 72 h), the animals were anesthetized and killed for the follow-up experiment.

### Neuron Culture

Newly postnatal C57BL/6J mice were purchased from the Department of Laboratory Animal Science, Peking University Health Science Center, Beijing. Anesthetized mice were killed by cervical dislocation. All tissues were maintained in D-Hank's solution (KCl.4 g, KH_2_PO_4_.06 g, NaCl 8.0 g, NaHCO_3_ 0.35 g, Na_2_HPO_4_·12H_2_O.132 g, D-Glucose 1.0 g in 1 L dddH_2_O, pH 7.4) and chilled on ice. The dissected medial prefrontal cortex was centrifuged (1,000 r/min, 7 min, room temperature) after dispersed by trituration and 0.25% trypsin digestion (Hyclone. 5 min, 37°C). The culture medium was DMEM (Gibco, United States) supplemented to 10% FBS (Gibco, United States) for the first 4 h, then changed to neurobasal (Gibco, United States) with 2% B27 (Gibco, United States) and 0.5 mM L-glutamine (Sigma-Aldrich, United States). Cells were plated on 0.1% poly-L-lysine (Sigma-Aldrich, United States) coated 6-well plates at 1 × 10^6^ cells/ml of medium, and maintained in an incubator at 37°C and 5% CO_2_. Urea (Sigma-Aldrich, United States), mannitol (Sigma-Aldrich, United States), and sucrose (Sigma-Aldrich, United States) were dissolved into the medium for stimulation. Neurons were subjected to subsequent experiments after 9 days culture.

### Human Subjects

All human sample studies were conducted in the Second Affiliated Hospital of Chongqing Medical University, with the approval of the Medical Research Ethics Committee and written informed consent. The material we used was post-mortem material. Forensics used part of the cerebral cortex to make paraffin sections. We performed antigen retrieval and immunofluorescence staining.

### Immunofluorescence

The mice or rats were deeply anesthetized with pentobarbital (85 mg/kg, i.p.) and perfusion with 20 ml of 0.9% saline, followed by fresh 4% paraformaldehyde in 0.01 M phosphate-buffered saline (pH 7.3). After perfusion, brains were taken out for an additional fixation overnight in 4% paraformaldehyde, followed by a graded series of sucrose-gradient dehydration. Dehydrated samples were frozen and sectioned in the coronal plane at 20 μm for immunofluorescent detection (Leica, Germany). The human brain slices were paraffin sections and needed antigen retrieval. Slides were processed for antigen retrieval and were dipped in 0.1 M sodium citrate buffer (pH 6.0) and heated to boiling for 5 min twice in a Microwave oven and left to return to ambient temperature. The brain slices were subsequently washed three times by phosphate buffer saline (PBS) for 5 min. Then the slices were washed in PBS–T (0.3% Triton X−100 in PBS) for 0.5 h at 37°C and transferred to blocking solution (0.3% PBS–T in 5% goat serum) overnight at 4°C before ~36 h incubation (0.3% PBS–T in 1% serum) in primary antibody solution [rabbit anti–UT-B with 1:100 working dilution, a gift from Dr. Trinh-Trang-Tan MM (Trinh-Trang-Tan et al., 2002); microtubule-associated protein 2 (MAP2) with 1:200 working dilution, abcam, United Kingdom; GM130 with 1:200 working dilution, BD, United States; PSD95 with 1:200 working dilution, CST, United States; Synapsin1 with 1:200 working dilution, abcam, United Kingdom]. Slices were then washed in PBS thrice and incubated for 0.5 h with secondary antibody (Cy3 goat anti-rabbit IgG 1:200, Invitrogen; Alex Fluor 488 goat anti-mouse IgG 1:200, Invitrogen, Italy; Hoechst 1:1000, Leagene, China) for fluorescent detection. Fluorescent image acquisition was performed with a Leica TCS SP8 confocal laser microscope.

### Immunohistochemistry

Sections were deparaffinized in xylene and rehydrated through graded alcohols to water. Slides were dipped in 0.1 M sodium citrate buffer (pH 6.0) and heated to boiling for 5 min twice in a microwave oven and left to return to ambient temperature. And then the sections were incubated in 3% hydrogen peroxide for 10 min at room temperature to block endogenous peroxidase activity. Followed by incubation in 10% goat serum, the sections were incubated overnight at 4°C with the primary rabbit polyclonal anti-UT-B antibody with 1:500 working dilution. After rinsing with phosphate buffer solution for 5 min thrice, the sections were performed with a sensitive polymer-helper detection system (Polink-2 plus® Zhongshan Golden Bridge Biotechnology Co., Ltd., Xuanwu District, China) following the manufacturer's instruction. Diaminobenzidine was used for staining development and the sections were counterstained with hematoxylin and then examined by light microscopy. Negative controls consisted of substituting normal serum for primary antibodies. The mean optical density of immunostained slides for UT-B was analyzed using the Image-Pro Plus software (Media Cybernetics, Silver Springs, MD, USA). The slides were scanned using 20 × magnification. By comparing each time point, the tendency variations of UT-B expression can be obtained.

## Results

### UT-B Mainly Expressed in the Astrocytes and Interneurons

**Figure 1:**
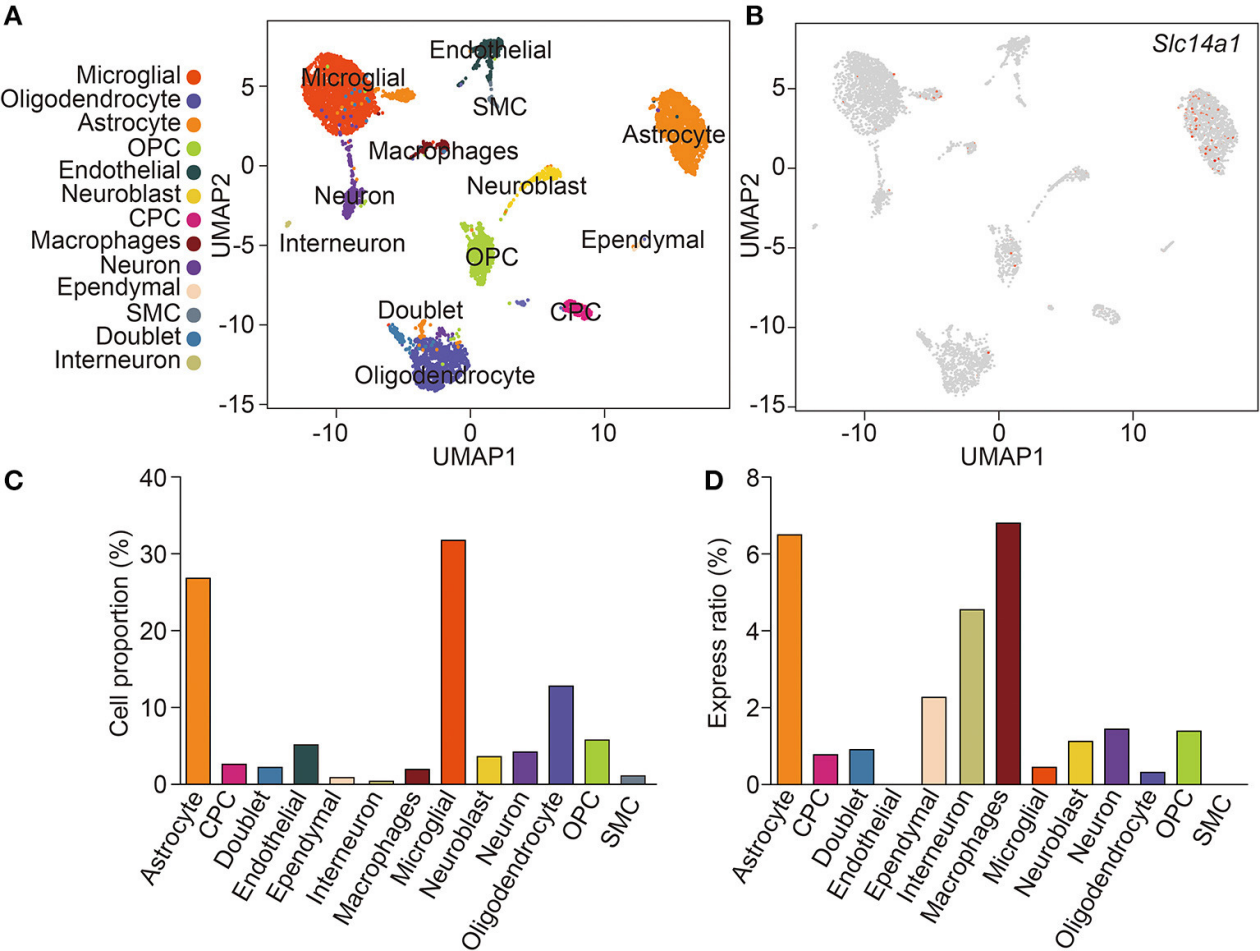
scRNA-seq analysis of wild-type mouse brain. **(A)** Unsupervised clustering uniform manifold approximation (UMAP) plot of CNS cell of wild-type mice. OPC, oligodendrocyte precursor cells; CPC, choroid plexus cells; SMC, smooth muscle cells (*n* = 4,884). **(B)** Expression scatterplots of *Slc14a1* in population from wild-type mice. **(C)** Cell proportion of CNS cell. **(D)** Express ratio analysis of *Slc14a1* based on percentage composition.

ScRNA-seq enabled the unbiased investigation of cells in complex tissues to identify novel populations and gene expression in development and disease (Tanay and Regev, 2017; Giladi and Amit, 2018). We collected brain tissues from adult wild-type mice and digested them to form a single-cell suspension, which obtained more than 4,884 cells. We used genome-wide single-cell gene expression profiling data to classify cell types. An unbiased iterative clustering analysis classified the cells as neuronal and non-neuronal cells. The non-neuronal cells are further segregated into glial types (microglia, astrocytes, oligodendrocyte precursor cells, and oligodendrocytes) and other cell types (smooth muscle cells, endothelial, choroid plexus cells, macrophages, dendritic cell, and ependymal cells) (Figure 1A). The markers used for each cell type were shown in Table 1. By focusing on cells expressing *Slc14a1*, we identified that *Slc14a1* was expressed in most cells except endothelial and smooth muscle cells (Figure 1B). Combined with the cell proportion analysis, the cells with the higher expression rate were astrocytes, neurons, macrophages, and dendritic cells (Figures 1C,D). To our surprise, the expression of UT-B was not only in the different types of glial cells, but also in the neurons These data suggested that *Slc14a1* was expressed in various cells in the brain, and its function needs to be further explored.

**Table 1:** All cell types were obtained by scRNA-seq analysis and their corresponding marker.

	**Marker**
Microglial cells	Cx3cr1, Siglech, P2ry12, Tmem119
Oligodendrocytes	Plp1, Cldn11, Mal, Mag, Mog, Mobp
Astrocytes	Htra1, Aqp4, Clu, Slc1a3, Aldoc
Oligodendrocytes precursor cells	Pdgfra, Olig1, Cspg4, Gpr17
Endothelial cells	Cldn5, Igfbp7, Pecam1
Neuroblasts	Sox11, Neurod1, Ccdn2, Dlx1
Choroid plexus cells	Ttr, Kl, Sostdc1
Macrophages	Cd163, Mrc1, Lyz2
Dendritic cells (DCs)	H2-ab1, Cd209a
Neurons	Snap25, Nrgn, Meg3
Ependymal cells	Ccdc153, Rarres2, Tmem212
Smooth muscle cells (SMC)	Acta2, Myl9, Rgs5
Interneurons	Ndnf, Reln, Lhx1

*scRNA-seq, single-cell RNA sequencing*.

### Expression of UT-B in Glial Cells

**Figure 2:**
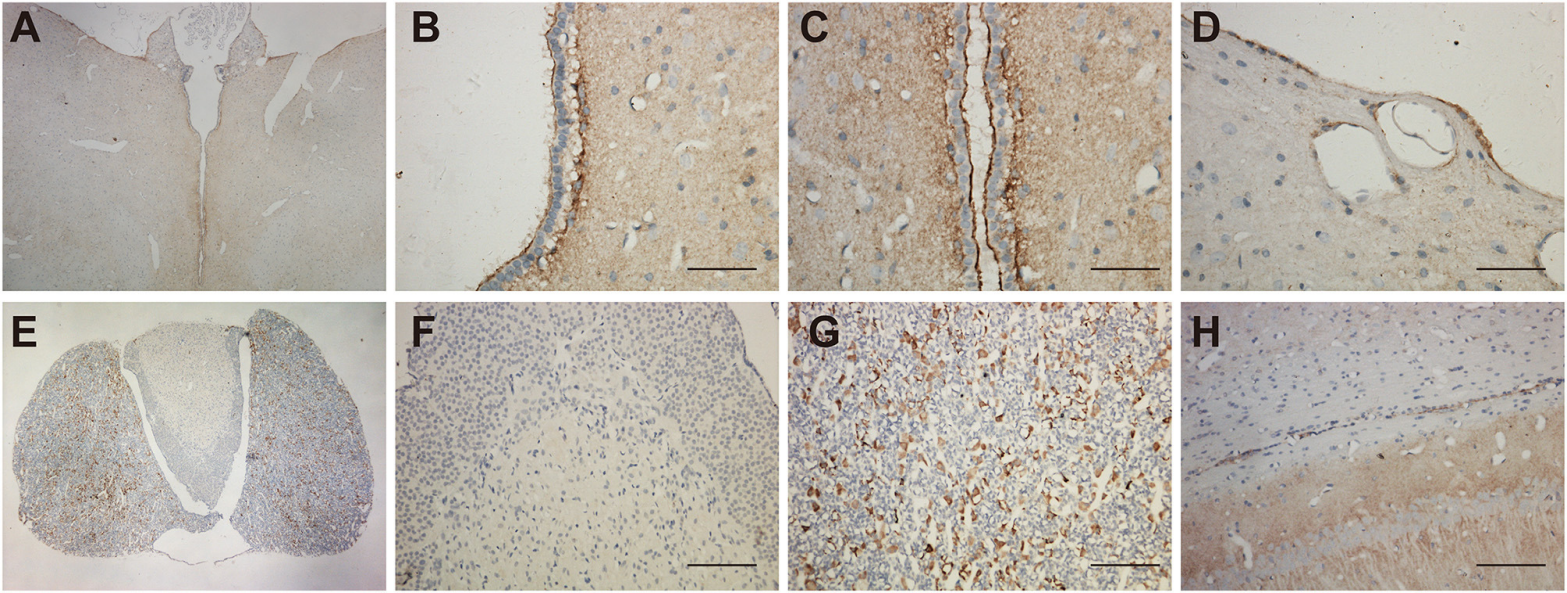
Distribution of UT-B in the third ventricular and neurohypophysis by immunohistochemistry. **(A)** UT-B was expressed around the third ventricular wall. **(B,C)** High magnification view of the third ventricle, strong UT-B labeling is present in the subependymal glia limitans, and free surface of ependymal cells. **(D)** Cerebral pia mater. **(E)** Low magnification of the hypophysis illustrating that UT-B is only expressed in the adenohypophysis. **(F)** High magnification view of the neurohypophysis. **(G)** High magnification view of the adenohypophysis. **(H)** UT-B by glial cells in white-matter fiber tracts. Scale bar, 200 μm. UT-B, urea transporter B.

Morphological experiments were conducted to verify scRNA-seq results. The immunoreactivity was found in astrocytes, but not in the endothelial cells of blood vessels in the brain. For the brain region, we observed the UT-B expression near the ventricle, hypophysis and hippocampus. UT-B was distinctly and strongly expressed at the third cerebral ventricular wall (Figure 2A). Glia limitans consisted of astrocytes that separated the brain tissue from the surrounding subependymal and subpial space. Higher magnification of the third cerebral ventricles (Figures 2B,C) had more sweeping views that exhibited that UT-B signals were detected along the glia limitans bilaterally and free surface of ependymal cells. Furthermore, UT-B-positive cells were observed in the cerebral pia mater, covering the brain tissue (Figure 2D). In hypophysis (Figure 2E), the distribution of UT-B showed a tremendous different modality from other parts; No UT-B-positive glial cells were observed in neurohypophysis at all (Figure 2F). In contrast, a marked expression of UT-B was observed in the adenohypophysis (Figure 2G). UT-B positive glial cells in white-matter fiber tracts (Figure 2H) were found too.

**Figure 3:**
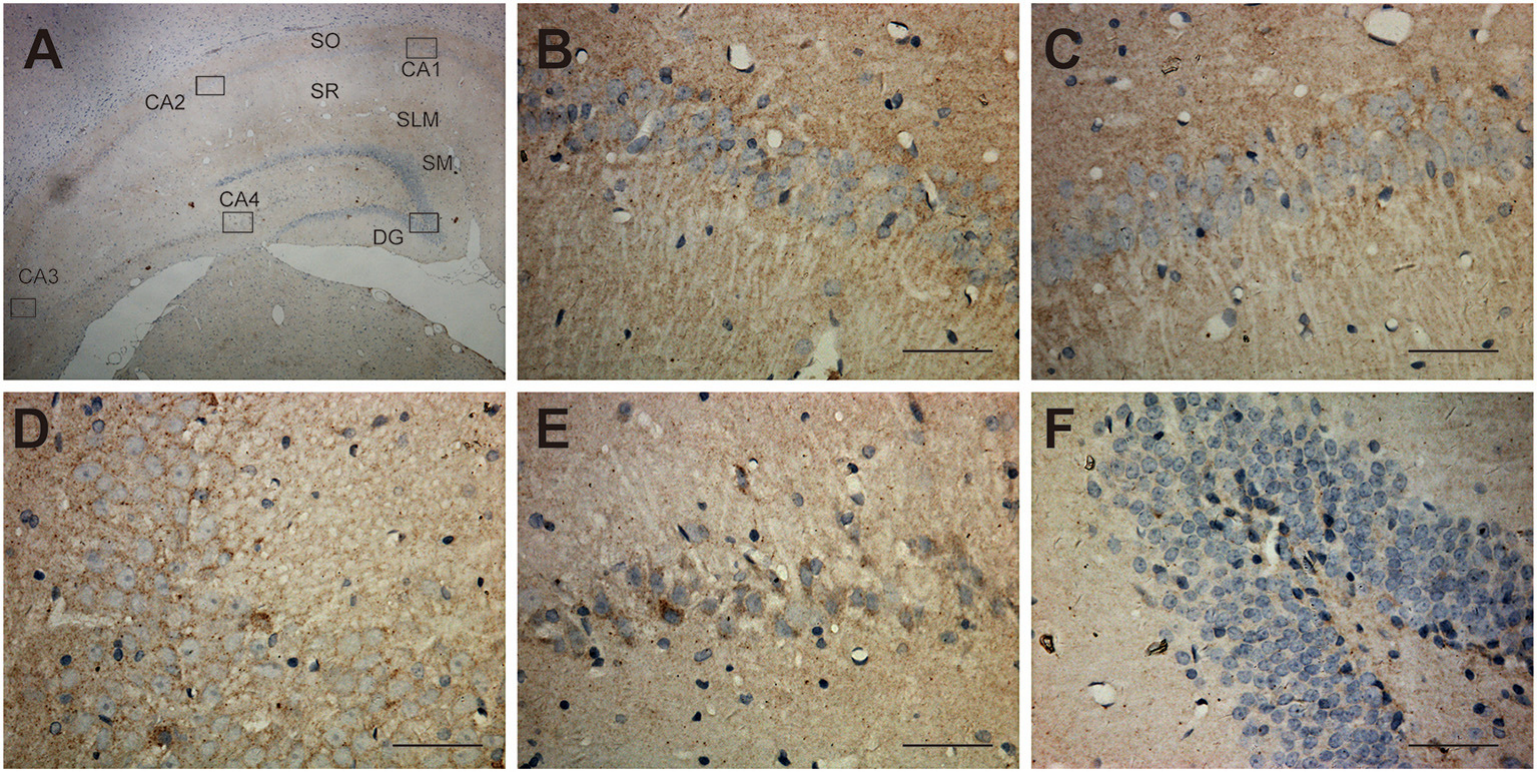
Distribution of UT-B in the hippocampus detected by immunohistochemistry. **(A)** UT-B is widely distributed throughout the hippocampus. **(B–E)** High magnification view of CA1-4: UT-B signals were not significantly observed at CA1 or CA2 but were gradually enhanced by astrocytes at CA3 and CA4. **(F)** High magnification view of dentate gyrus: UT-B signals by astrocytes in the granule cell layer in the dentate gyrus. SO, stratum oriens; SR, stratum radiatum; SLM, stratum lacunosum-moleculare; SM, molecular layer; UT-B, urea transporter B. Scale bar, 200 μm.

Shown in the cross-section of the hippocampus, UT-B IHC of hippocampal formation shows layer-specific expression. In lower magnification (Figure 3A), the UT-B signals reside in the granule cell layer in the dentate gyrus and stratum oriens (so) and distal region of stratum lacunosum-moleculare (slm) of the CA1 region. In higher magnification of each region, the UT-B signals were not significantly observed in the cytoplasm or perinuclear of any cell but in the fibers at CA1 (Figure 3B) or CA2 (Figure 3C). Nevertheless, the UT-B signals were gradually enhanced by astrocytes at CA3 (Figure 3D) and CA4 (Figure 3E). We observed the strongest signal in some astrocytes in the granular cell layer of the dentate gyrus (Figure 3F).

### Expression of UT-B in Neurons of the Central Nervous System

**Figure 4:**
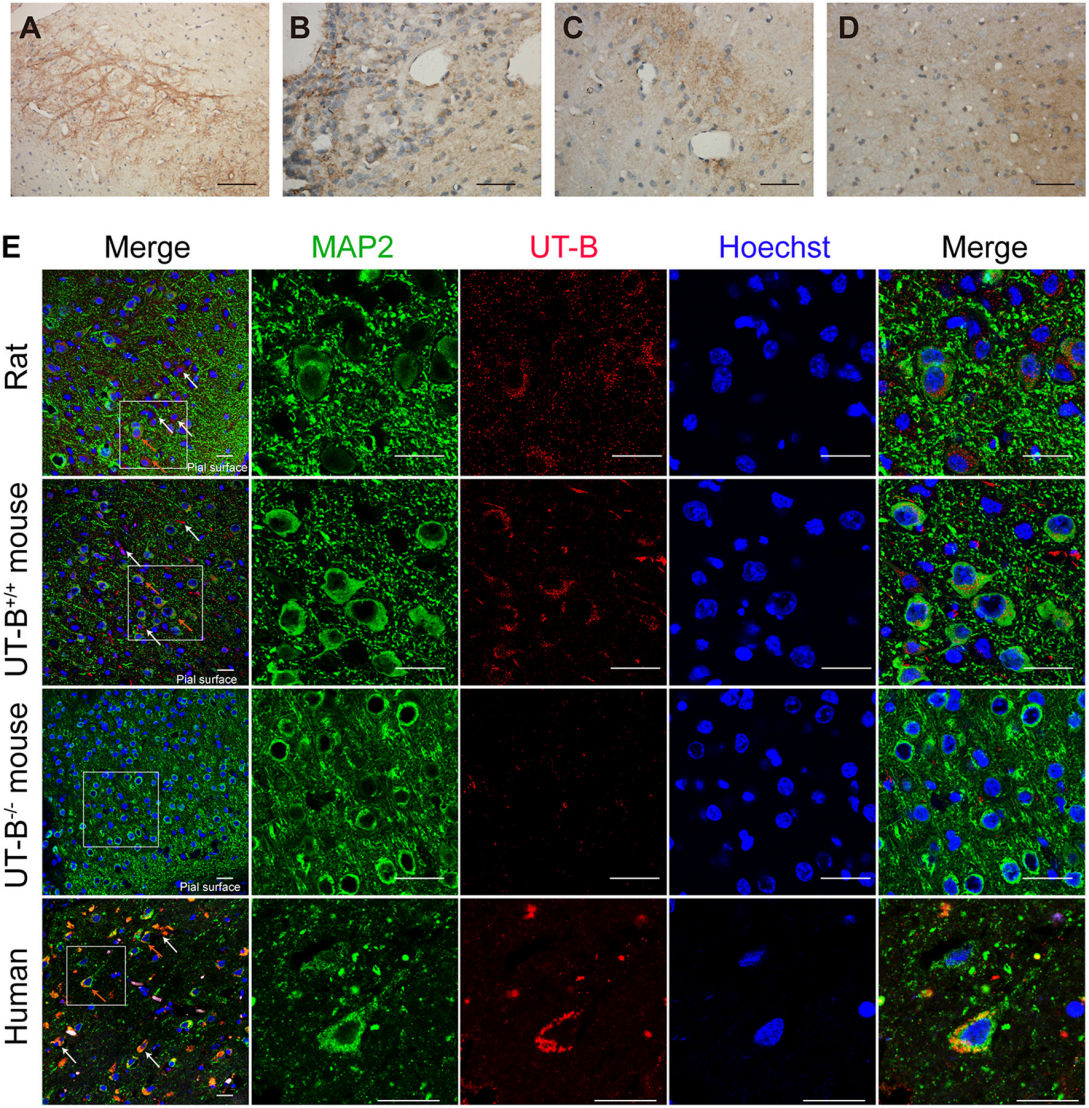
UT-B is expressed in neurons. **(a–d)** Immunohistochemistry of substantia nigra, habenula, the lateral inferior nucleus of thalamus and cortex. Scale bar, 200 μm. **(e)** Immunofluorescence revealed that UT-B (red) was expressed in MAP2-positive neurons (green) in rats, wild-type mice, UT-B knockout mice, and the human cerebral cortex. The cells indicated by the white arrows and the red stripe staining are positively stained astrocytes, and the orange arrows are the positively stained neurons. The pial surface is located in the lower right corner of the image. The large-field images of rats and mice mainly show the expression of UT-B in the three layers of the cerebral cortex, including molecular layer, external granule cell layer, and external pyramidal cell layer. Scale bar, 50 μm. UT-B, urea transporter B.

The expression of UT-B in the brain was not restricted to glial cells but also a select subgroup of neurons. The UT-B-positive neurons could be distinguished from astrocytes based on their larger size and more intense labeling. The subgroup of neurons in substantia nigra (Figure 4A), habenula (Figure 4B), the lateral inferior nucleus of the thalamus (Figure 4C), and cerebral cortex (Figures 4D,E) were labeled with UT-B, which had distinct contrast with ambient. The rambling funicular appearance of immunoreactivity was unique in substantia nigra (Figure 4A). Both glial cells and neurons were labeled in habenula (Figure 4B). Further, we detected UT-B expression in the cerebral cortex of different species, including mice, rats, and humans. The co-staining of UT-B and MAP2, a neuron-specific marker, suggested that UT-B was expressed in partial neurons in the cerebral cortex (indicated by orange arrows). The UT-B knockout mice were used to ensure the reliability of immunofluorescence (Figure 4D). In lower magnification, the pial surface is located in the lower right corner of the image. UT-B positively stained neurons were distributed in the molecular layer, external granule cell layer, and external pyramidal cell layer. In addition, as in previous studies (Trinh-Trang-Tan et al., 2002), we also observed the expression of UT-B in glial cells (indicated by white arrows).

### Subcellular Localization of UT-B in Normal Circumstance, Hypo- or Hyperosmolar State

**Figure 5:**
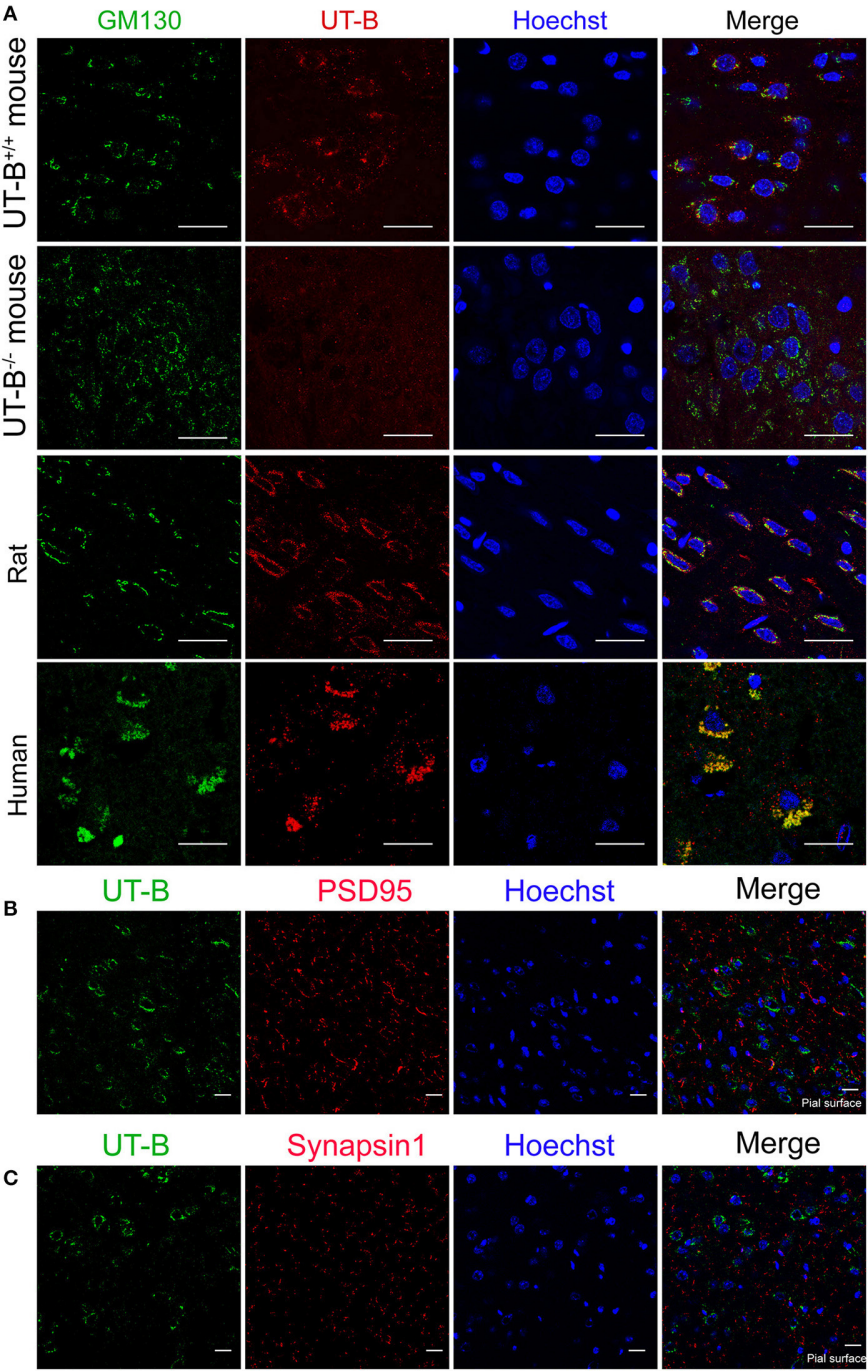
UT-B is located on the Golgi apparatus in normal circumstances. **(A)** Representative immunofluorescence images of wild-type rats, wild-type mice, UT-B knockout mice, and human cerebral cortex revealed colocalization of UT-B (red) and GM130 (green). Nuclei were stained blue with Hoechst. **(B)** Co-stained immunofluorescence images of UT-B (green) and postsynaptic membrane marker PSD95 (red) in the cerebral cortex of wild-type mice. **(C)** Co-stained immunofluorescence images of UT-B (green) and synaptic marker synapsin 1(red) in the cerebral cortex of wild-type mice. The pial surface is located in the lower right corner of the image. The images of b and c mainly show the internal granule cell layer and the internal pyramidal cell layer. Scale bar, 50 μm. UT-B, urea transporter B.

The location of UT-B at the Golgi apparatus was assessed. We co-stained UT-B and GM130, an established Golgi apparatus membrane protein. Immunofluorescence of the mice brain slices showed that UT-B is physiologically located in the Golgi apparatus. The same result was also observed in rat and human brain slices (Figure 5A).

We performed the co-staining of neuronal synaptic markers and UT-B in the cerebral cortex of wild-type mice (Figures 5B,C). There was no co-expression of UT-B and synaptic markers PSD95 or synapsin1. The results show that UT-B is not expressed on the synapses of neurons under normal physiological conditions.

**Figure 6:**
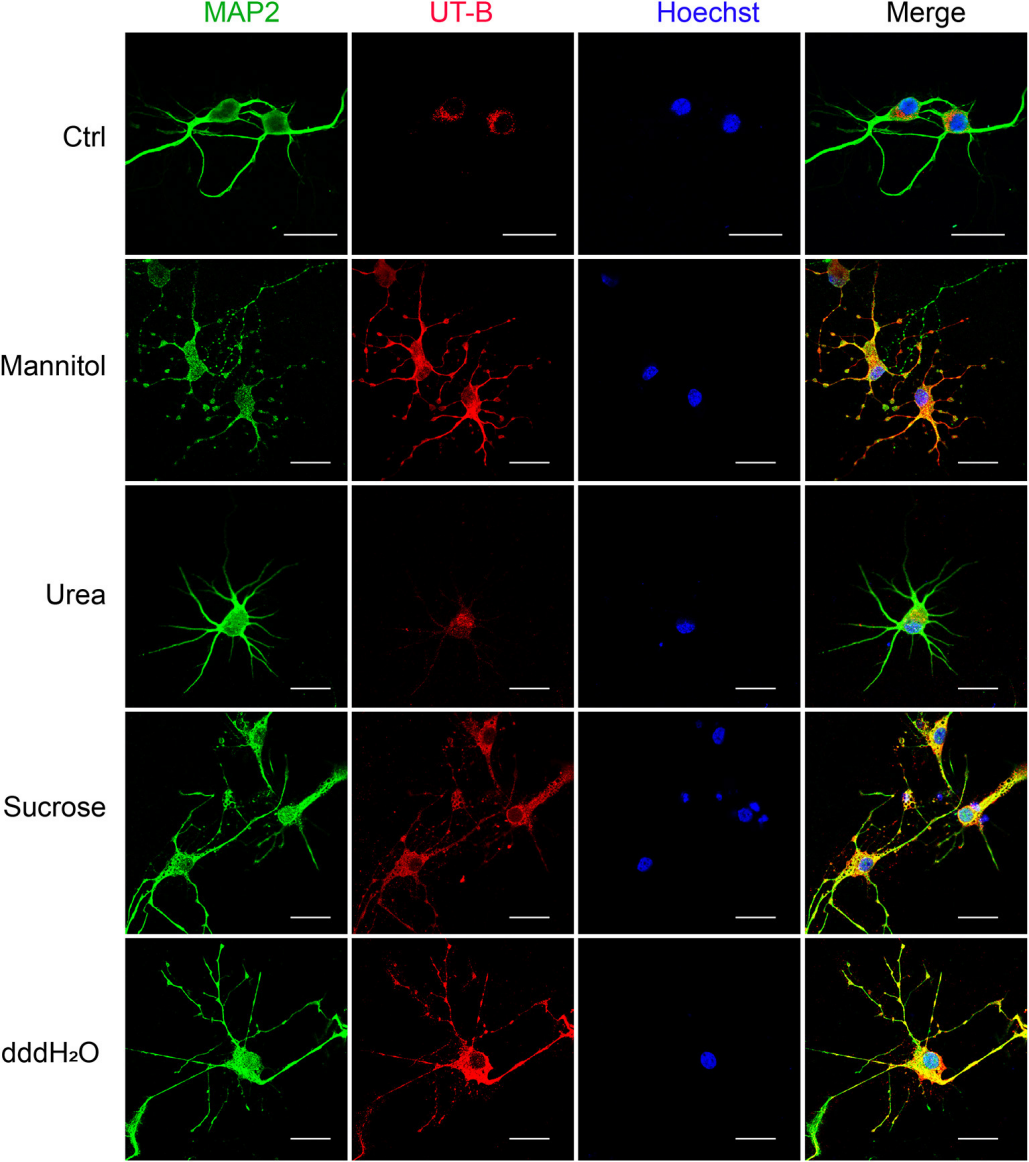
UT-B translocation in hypo- or hyperosmolar condition. Representative immunofluorescence revealed that UT-B (red) expression level increased in MAP2-positive neurons (green) under 60 mM mannitol, sucrose, and 1/4 of the total volume of water. But no change was observed under urea stimulation. Scale bar, 50 μm. UT-B, urea transporter B.

Since UT-B transports both water and urea, it plays a vital role in maintaining the osmotic pressure in cells. Therefore, experiments were designed to assess the expression regulation of UT-B under different osmotic pressure. We cultured primary cortical neurons from wild-type mice, added 60 mM mannitol, urea, sucrose, or 1/4 culture medium volume of the dddH_2_O, respectively, for 24 h. We found that under the stimulation of sucrose and mannitol, UT-B translocated to neurites with increased expression (Figure 6). Interestingly, the above changes did not occur in the case of urea stimulation. The possible reason was that UT-B expressed by neurons transported urea rapidly, making the internal and external osmotic pressure consistent. Under the condition of hypo-osmotic pressure, the expression and location of UT-B were also altered (Figure 6), indicating that UT-B is bidirectionally regulated by osmotic pressure.

### Expression of UT-B Increased After Traumatic Brain Injury

**Figure 7:**
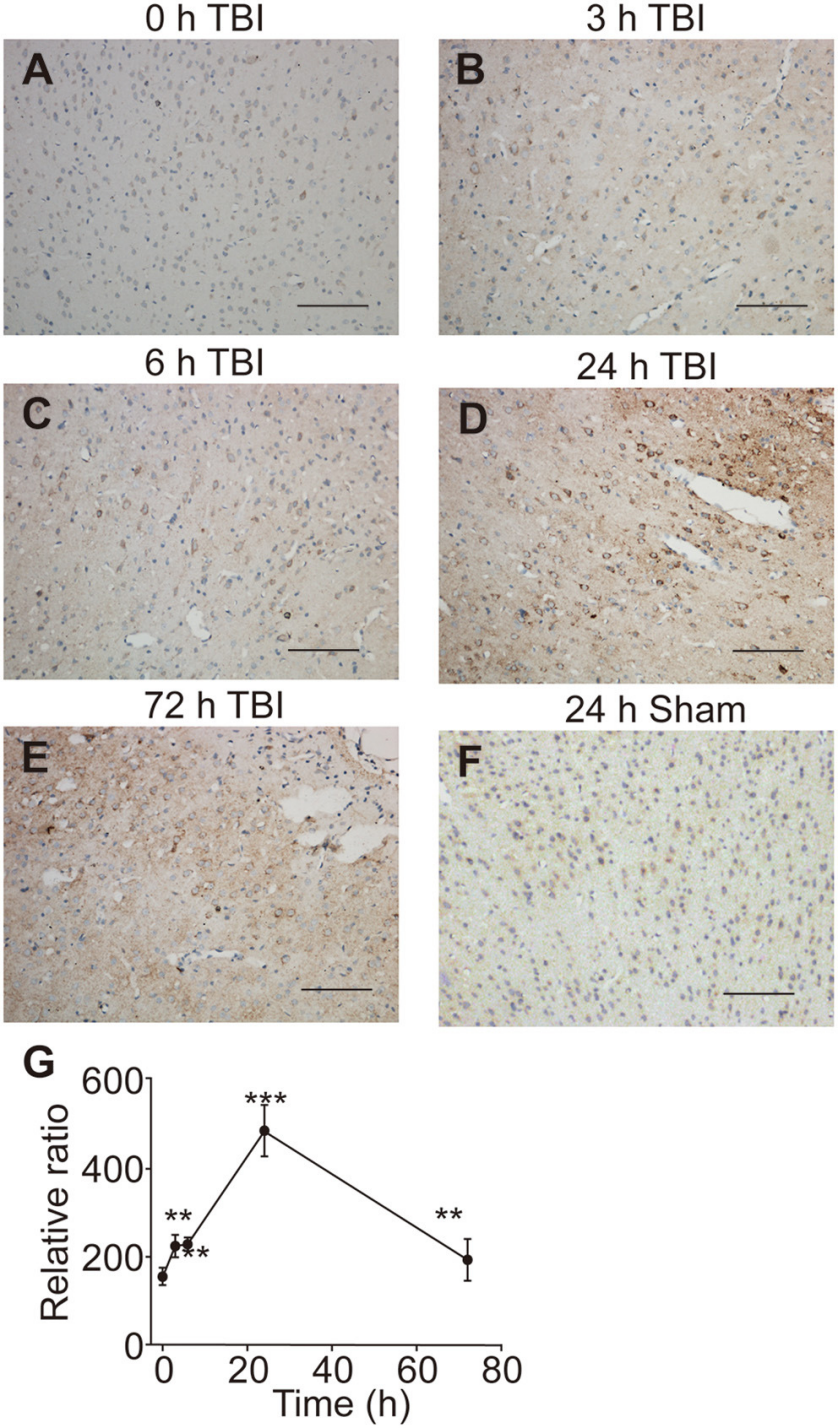
UT-B expression before and after traumatic injury detected by immunohistochemistry. **(A)** Immunohistochemistry of UT-B in the ipsilateral cortex near the site of before injury. **(B–E)** Immunohistochemistry of UT-B in the ipsilateral cortex near the site of injury after 3, 6, 24, and 72 h. **(F)** Immunohistochemistry of UT-B of the sham operation group mice in the ipsilateral cortex near the site after 24 h. **(G)** The tendency of UT-B level analyzed by Image-pro Plus. Data presented as mean ± SEM. **P < 0.01, ***P < 0.001, *n* = 6. UT-B, urea transporter B.

Since the UT-B was regulated by osmotic pressure, we hypothesized that the expression of UT-B changed in brain edema. The traumatic brain edema model was employed because it is a combined cellular and vasogenic brain edema. We assessed the signals of UT-B in the ipsilateral cortex near the site of injury right before (Figure 7A), 3 h (Figure 7B), 6 h (Figure 7C), 24 h (Figure 7D), or 72 h (Figure 7E) after traumatic brain injury model (TBI) by immunohistochemistry. The rats in the sham operation group were just cut off the scalp and created a bone window after anesthesia, without any blows. There was no significant change in the expression of UT-B in the sham group 24 h after operation (Figure 7F). At the same layer, the number of UT-B-positive neurons in the TBI rats had been elevating with time going on, reaching a peak at 24 h post-trauma then it began decreasing. Meanwhile, the intensity of immunoreactivity had been changing with the number of UT-B-positive neurons proportionally (Figure 7G).

## Discussion

The current study revealed the precise distribution of UT-B in the brains of mice, rats, and humans. We mainly demonstrated that UT-B is expressed extensively both in neurons and glial cells in the central nervous system. Moreover, the results of immunohistochemistry and scRNA-seq together proved that UT-B was highly expressed in ependymal cells and glia limitans. UT-B is also expressed by neurons in the cerebral cortex, substantia nigra, habenula, lateral inferior nucleus of the thalamus, and by glial cells in the hippocampus as well as in the white-matter fiber tracts.

The previous study displayed that the UT-B was found in astrocytes in the granule cell layer in the dentate gyrus and throughout the other parts of the hippocampal formation based on *in situ* hybridization (Doran et al., 2006). Indeed, unlike renal descending *vasa recta* (Pallone, 1994) and endothelia in a blood vessel (Wagner et al., 2002), UT-B was not expressed in endothelial cells of cerebral blood vessels. Contrary to previous results (Berger et al., 1998; Trinh-Trang-Tan et al., 2002), scRNA-seq data showed that UT-B was expressed in the choroid plexus cell although the expression level was extremely low, which needed more data to confirm. Interestingly, even though the previous study (Berger et al., 1998) repeatedly emphasized that the UT-B was tested by the glial cells in the cerebral cortex, nonetheless we found that neurons also expressed UT-B through scRNA-seq and immunofluorescence. It is worth noting that UT-B was not expressed in all kinds of neurons. ScRNA-seq data showed that neurons expressing UT-B were mainly concentrated in neurons labeled with Ndnf, Reln, and Lhx1. However, the proportion of positive cells was too low. We need larger cell samples to explore the subtypes of neurons or the common markers of neurons with UT-B expression.

In mammals, the major biochemical for the disposal of waste nitrogen is the urea cycle which ends up producing urea (Felig, 1975; Zhang et al., 2018). Active argininosuccinate synthetase, argininosuccinase, and arginase namely enzymes of the latter half of the urea cycle exist in brain tissue (Ratner et al., 1960), suggesting that cells in the brain also produce urea. Based on this hypothesis, we infer that the UT-B expressed by astrocytes near the vessels, by cells around the third ventricle, and in the cerebral pia mater plays a crucial role in facilitating the transport of urea into the blood or cerebrospinal fluid. In UT-B knockout mice, urea accumulation in the brain results in depressive-like behavior (Li et al., 2011; Wang et al., 2019), which also supports this inference. The substantia nigra staining is different from the cell staining. UT-B is highly expressed in the substantia nigra, and the neurites are positively stained, while UT-B is only expressed in the Golgi, but not synapses under physiological conditions in other neurons. High expression of UT-B in the habenula, substantia nigra, and lateral inferior nucleus of the thalamus may be a bridge between their unique metabolism and unique function. Habenula is associated with the etiology of depressive disorder (Sartorius et al., 2010; Yang L. M. et al., 2014). Besides substantia nigra, pars compacta are associated with Parkinson's disease and other common tremor disorders (Homayoon et al., 2019). We infer that urea is a by-product of some unknown metabolic processes that maintain the normal function of those unique sites in the brain.

Previous work showed that urea permeability was stimulated by hyperosmolarity in the form of effective osmoles (sodium chloride, mannitol) but not ineffective osmoles (urea) in isolated perfused tubules (Sands and Schrader, 1991). In neurons, the expression of UT-B was up-regulated in response to hyperosmolarity in the form of mannitol and sucrose but not urea. The low osmosis caused by water also induced the high expression of UT-B. We speculated that it was a compensatory increase for the transfer of water. UT-B also acts as a water channel, and the single-channel (per molecule) water permeability of UT-B in erythrocytes is very similar to that of AQP1 (Yang and Verkman, 2002). Although AQP4 mediates water transport in the brain, it is not expressed in neurons. That is to say, UT-B could be the essential water channel in neurons.

AQP4 deletion alleviates cytotoxic brain edema but aggravates vasogenic brain edema (Papadopoulos and Verkman, 2007; Yang et al., 2008). Since AQP4 and UT-B had very similar brain distribution, we established models of cytotoxic and vasogenic brain edema in UT-B knockout mice. There was no difference in the survival rate, neurological score, or brain water content between UT-B knockout mice and wild-type mice. This suggests that AQP4 plays a more dominant role in brain water transport than UT-B. However, neurons express UT-B but not AQP4, which indicates that UT-B may be involved in white matter diseases.

TBI can cause activation of excitatory amino acids, calcium overload, mitochondrial damage, activation of oxygen free radicals, activation of inflammatory system, etc. (Desai and Jain, 2018). These pathophysiological processes further lead to short-term or long-term secondary neuronal damage accompanied by cognitive, mental, and behavioral changes (Konigs et al., 2016). Urea is a metabolic end product is one of the factors leading to these adverse reactions (Li et al., 2011). In acute TBI, the urea accumulation in the brain is as follows: (1) Impaired blood circulation causes central regulation and autonomic nerve function damage, the protein catabolism in the stress process is correspondingly increased; (2) Meanwhile, a high dose of a dehydrating agent was used in the treatment to concentrate the cerebrospinal fluid which could increase the concentration of urea. Studies have reported that polyamines are rapidly synthesized after TBI in both humans (Timothy et al., 1996) and rodents (Zahedi et al., 2010). The brain has complete metabolism pathways for synthesizing polyamines (Bolkenius and Seiler, 1986). Ornithine is also a product of the urea cycle from arginine (Morris, 2004), so polyamine synthesis can reflect the formation of urea. After various neuronal impairments, polyamines display a durable power of protective function, suggesting a powerful feature of neuron regeneration (Dornay et al., 1986; Gilad and Gilad, 1992). A previous study has found that ornithine decarboxylase increases six- and three-fold, respectively, at 6 and 24 h after brain injury and returns to control levels at 72 h (Raghavendra Rao et al., 1998). Zahedi et al. found that spermine levels were normal at 6 h and decreased slightly at 24 h, but were normal by 72 h post-injury (Zahedi et al., 2010). Thus, we infer that variation of UT-B expression in the ipsilateral cortex near the site of traumatic injury is an adaptive modulation for constantly changing the urea level.

In conclusion, we found that UT-B is selectively expressed in the central nervous system glial cells and neurons; and the translocation of UT-B in hypo- or hyperosmolar conditions. The expression is highly consistent in mice, rats, and humans, proving that UT-B is a very conservative urea transporter. We revealed that UT-B changed among time after TBI, and the turning point is 24 h after injury, which may be adaptive adjustments to the changed urea levels. However, the molecular mechanism by which UT-B exerts specific effects in various brain regions and post-traumatic brain injury is unclear. Hence, further research is needed to thoroughly elucidate the role and mechanism of UT-B in the central nervous system.

## Data Availability Statement

The raw data supporting the conclusions of this article will be made available by the authors, without undue reservation, to any qualified researcher.

## Ethics Statement

The studies involving human participants were reviewed and approved by the Second Affiliated Hospital of Chongqing Medical University. Written informed consent for participation was not required for this study in accordance with the national legislation and the institutional requirements. The animal study was reviewed and approved by Animal studies were conducted in accordance with the procedure approved by the Animal Ethics Committee of Peking University, the approval number is LA2020354.

## Author Contributions

BH, HW, JR, and BY conceptualized the project. BH and HW were involved in designing and conducting the experiments. DZ and JM assisted with cell culture. ML performed part of animal experiments. BH analyzed data and prepared the draft with input from all authors. JR and BY supervised research. All authors contributed to the article and approved the submitted version.

## Conflict of Interest

The authors declare that the research was conducted in the absence of any commercial or financial relationships that could be construed as a potential conflict of interest.
